# A new paradigm of learned cooperation reveals extensive social coordination and specific cortical activation in mice

**DOI:** 10.1186/s13041-023-01032-y

**Published:** 2023-05-11

**Authors:** Ke-Ming Zhang, Yan Shen, Chun-Hui Jia, Hao Wang, Guo-Qiang Bi, Pak-Ming Lau

**Affiliations:** 1 CAS Key Laboratory of Brain Function and Disease, School of Life Sciences, University of Science and Technology of China, 230026 Hefei, China; 2 Interdisciplinary Center for Brain Information, The Brain Cognition and Brain Disease Institute, Shenzhen Institute of Advanced Technology, Chinese Academy of Sciences, Shenzhen, 518055 China; 3 National Engineering Laboratory for Brain-Inspired Intelligence Technology and Application, School of Information Science and Technology, University of Science and Technology of China, 230026 Hefei, China; 4 Institute of Artificial Intelligence, Hefei Comprehensive National Science Center, Hefei, 230088 China

**Keywords:** Cooperation, Social interaction, c-Fos, Neuronal activity trace, VISoR

## Abstract

Cooperation is a social behavior crucial for the survival of many species, including humans. Several experimental paradigms have been established to study cooperative behavior and related neural activity in different animal species. Although mice exhibit limited cooperative capacity in some behavioral paradigms, it is still interesting to explore their cooperative behavior and the underlying neural mechanisms. Here, we developed a new paradigm for training and testing cooperative behavior in mice based on coordinated lever-pressing and analyzed social interactions between the animals during cooperation. We observed extensive social contact and waiting behavior in cooperating animals, with the number of such events positively correlated with the success of cooperation. Using c-Fos immunostaining and a high-speed volumetric imaging with synchronized on-the-fly scan and readout (VISoR) system, we further mapped whole-brain neuronal activity trace following cooperation. Significantly higher levels of c-Fos expression were observed in cortical areas including the frontal pole, motor cortex, anterior cingulate area, and prelimbic area. These observations highlight social interaction and coordination in cooperative behavior and provide clues for further study of the underlying neural circuitry mechanisms.

**Supplementary Information:**

The online version contains supplementary material available at 10.1186/s13041-023-01032-y.

## Main text

Cooperation, in which multiple participants act together for mutual benefits [1], is critical to the survival and evolution of many species, including humans [1, 2]. In the laboratory, animals can also learn to accomplish cooperative tasks. Monkeys can learn to cooperatively control the movement of a cursor on a screen [3], and rodents can learn tasks such as coordinated shuttling and nose-poking [4–6]. Although mice demonstrate relatively less cooperation [6], they do exhibit various prosocial behaviors [7–9]. Considering the abundance of transgenic mice available for recording and manipulating the activity of specific neuronal populations [10], it is worthwhile to explore the cooperation capability in mice. In this study, we trained mice to learn coordinated lever-pressing and then examined their social interactions during this cooperative behavior. We further evaluated potential brain circuits involved in cooperation with whole-brain imaging of c-Fos expression.

**Fig. 1:**
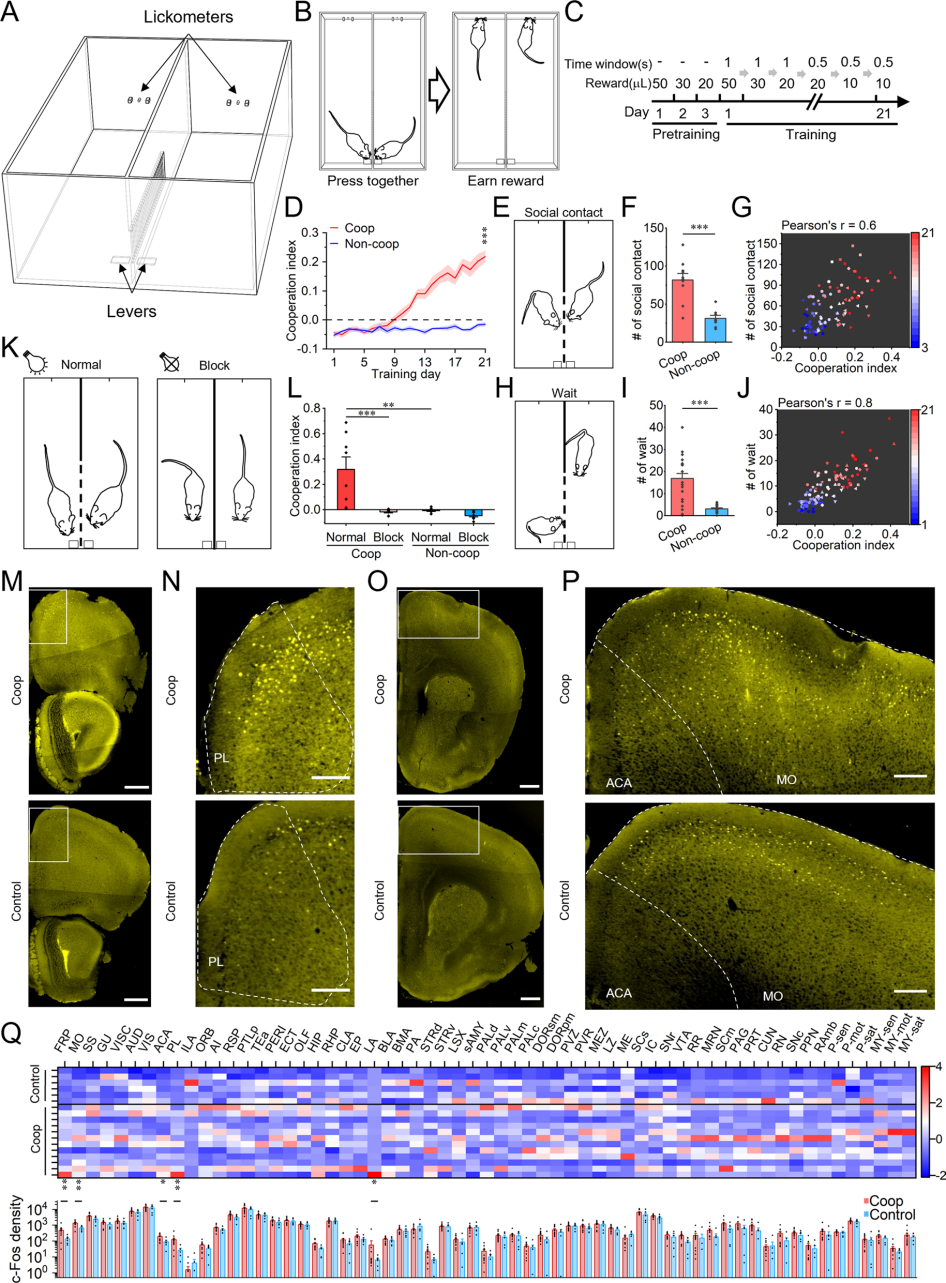
Cooperative behavior and related brain activity trace. **A** Schematic diagram of the experimental box for cooperation training and testing. **B** Cooperation requirement in the behavioral paradigm: the two mice need to press levers synchronously to obtain subsequent rewards. **C** Training schedule; reward value and time window of joint pressing for reward were changed according to criteria in Additional file 1: Fig. S1A. **D** Gradual increase of the cooperation index (CI) with training. The CI in the cooperative group (n = 20) was significantly greater than that in the non-cooperative group (n = 16) on the last day of training. ***p < 0.001; Mann–Whitney test. **E** Schematic diagram of social contact.** F** Social contact number of the coop group (mean of each pair of coop group during training days 17, 19 and 21, n = 10 pairs) was higher than that of the non-coop group (mean of each pair of non-coop group during training days 17, 19 and 21, n = 8 pairs). One-way ANOVA test. **G** Social contact number was positively correlated with CI. Dots with different shapes indicate the data of ten training pairs on various training days. The heat level indicates the number of training days.** H** Schematic diagram of waiting behavior.** I** Wait number of the coop group (mean of each mouse of the coop group during training days 17, 19 and 21, n = 20) was higher than that of the non-coop group (mean of each mouse of the non-coop group during training days 17, 19 and 21, n = 16). Mann–Whitney test. **J** Waiting number was positively correlated with CI. Dots with different shapes indicate the data of ten training pairs (mean waiting number of two mice) on various training days. The heat level indicates the number of training days.** K** Schematic diagram of the blocking social contact test. The partition wall was windowed, and the illumination was on under normal condition (left). The partition wall was unwindowed, and the illumination was off under the block condition (right). **L** Blocking social contact impaired cooperative behavior. Paired t test (normal vs block of coop group, n = 8) or Mann–Whitney test (normal of coop group vs normal of non-coop group, n = 8). **M, O** c-Fos expression of the coop and non-coop groups in different coronal sections (50 μm thick) of Bregma 2.96 mm (**M**) and 1.42 mm (**O**). Scale bar = 500 μm. **N, P** Enlarged details of the rectangular area in **M** (**N**) and **O** (**P**). Scale bar = 200 μm. **Q** Brain-wide neuronal activity trace during cooperation. Top: c-Fos density for each brain region of each mouse, normalized across all mice using the z-score (color-coded). Bottom: mean c-Fos density of the coop and control groups. Linear regression models (Y = βX + α) were established to compare the difference in c-Fos density between the coop (n = 12) and control groups (n = 6). Shading or error bars indicate the SEM. *p < 0.05, **p < 0.01, ***p < 0.001

For training the mice to learn cooperation, we designed a training box that was divided into two chambers by a transparent windowed partition wall, with a lever and a lickometer on the two ends of each chamber (Fig. 1A). The mice were first trained individually to obtain water rewards after lever-pressing (Additional file 1: Fig. S1A–C). Then they were divided into cooperative and non-cooperative groups and trained differently (Additional file 1: Fig. S1A). Mice in the non-cooperative group continued to be trained individually for lever pressing, whereas mice in the cooperative group were trained in pairs and needed to press the levers synchronously within a 1 s or 0.5 s window to receive rewards (Fig. 1B, C and Additional file 1: Fig. S1A). Because mice could also show synchronous lever-pressing by chance, we shuffled the timing of lever-pressing and licking behaviors of each pair of mice, and computed the ratios of synchronous pressing and total pressing under 1000 shuffled conditions. We then define the cooperation index (CI) as the synchronous pressing ratio (the number of measured synchronous pressing over total pressing), subtracting the top 95th percentile of the “synchronous pressing” ratios from the shuffled data. The mean CI of the cooperative group was found to increase with training and was significantly higher than that of the non-cooperative group on the last training day (Fig. 1D, Additional file 2: Video S1 and Additional file 3: Video S2). To evaluate the stability of this learned cooperative behavior, we performed three different tests including (1) partner swapping test, (2) obstacle test, and (3) long-term memory test (see “Methods”). None of the manipulations significantly affected the resulting CI (Additional file 1: Fig. S1D–F). Thus, the mice were able to learn the task, resist interference and stably express cooperative behavior.

Social contact is known to be necessary for cooperation [4, 6, 11]. To test the importance of social contact in the present task, we used DeepLabCut [12] to identify the neck and body coordinates of the mice and found that the mice indeed tended to shuttle synchronously during cooperation (Additional file 1: Fig. S2A, B). The body locations of the two paired mice in the cooperative group exhibited more overlap than the non-cooperative group (Additional file 1: Fig. S2C), and were positively correlated with CI (Additional file 1: Fig. S2D). To further analyze social behaviors, we extracted social contact events based on neck distance and body angle (Fig. 1E, Additional file 1: Fig. S2E and Additional file 4: Video S3). We found that the number of social contact events in the cooperative group was greater than that in the non-cooperative group (Fig. 1F). Furthermore, the number of social contact events was positively correlated with the CI in the cooperative group (Fig. 1G). In addition to social contact, mice also showed voluntary waiting behavior, i.e., a mouse did not press the lever until his partner had also reached the press area (Fig. 1H, Additional file 1: S2A and Additional file 5: Video S4). The number of waiting events in the cooperative group was significantly greater than that in the non-cooperative group (Fig. 1I) and was positively correlated with CI (Fig. 1J).

To evaluate the necessity of communication between partners during this task, we paired cooperatively trained mice with non-trained partners. It is interesting to note that the trained mouse apparently waited for the non-trained mouse to come close to the lever, and then pressed its lever and shuttled to the water nozzle (Additional file 6: Video S5). This suggests that the trained mouse could use the partner’s location information to direct its action. However, in such tests where the trained mice did not have effective communication with their non-trained partners, the CI of the trained animals decreased significantly to almost chance level (Additional file 1: Fig. S3A). This indicates the importance of effective communication for the animals to achieve high-level performance in coordination tasks, consistent with previous studies using robotic rat partners for similar tests [6]. In parallel experiments, we turned off the illumination light during cooperation tests for pairs of trained mice. The CI decreased significantly compared to the normal light-on condition but was still significantly higher than that of non-trained animals (Additional file 1: Fig. S3B), suggesting that other sensory modalities such as auditory or olfactory systems could also contribute to the performance. Finally, when we used an unwindowed partition wall in the test box and turned off light to block all sensory information needed for social interaction during the cooperation test, the CI for the tested pairs decreased to near the chance level (Fig. 1K, L). These results suggest that the coordinated lever-pressing of the mice is likely a form of cooperation that requires social interaction and communication.

Cooperation requires individuals to coordinate actions with their partners, and could involve concerted activity of various brain circuits [3, 5, 6]. To identify such circuits involved in cooperation, we used a volumetric imaging with synchronized on-the-fly-scan and readout (VISoR) system for whole-brain imaging [13] to obtain brain-wide c-Fos expression [14] in mice performing cooperative or non-cooperative tasks (Fig. 1M–P and Additional file 1: Fig. S4). Of all 61 brain regions evaluated, 5 were found to exhibit significantly elevated c-Fos expression in the cooperative group (Fig. 1M–Q). These regions include the frontal pole (FRP), somatomotor areas (MO), anterior cingulate area (ACA), prelimbic area (PL) and lateral amygdala nucleus (LA), suggesting possible involvement of various functions, such as decision making, motor planning, socializing and emotions, in cooperative behavior [15].

It is noted that the c-Fos expression across different brain areas showed strong individual variability in the cooperation group. This may be due to the variability of spontaneous behaviors and mental states during the period when the mice performed the test. It could also reflect different strategies of cooperation. In the future, refined behavior analysis and real-time neural activity recording are needed for further in-depth investigation. In the current study, we established an efficient paradigm of cooperative behavior in mice based on synchronized lever-pressing and revealed the relationship between characteristic social behaviors and cooperation. Together with brain-wide activity trace mapping, our work provides useful tools and clues for further studies of the neural mechanisms underlying cooperative behavior and its development through learning.

## Supplementary Information

**Additional file 1: Fig. S1.** Mice were able to learn the cooperative lever-pressing task. A Schematic diagram of the cooperative training procedure. B, C Mice were able to learn the individual lever-pressing task. B The total press number during pretraining increased. There was no significant difference between the coop and non-coop groups across the 3 pretraining days. One-way ANOVA test. C The total lick number during pretraining increased. There was no significant difference between the coop and non-coop groups across the 3 pretraining days. One-way ANOVA test. D Swapping partners did not affect the cooperation index. The CI of mice cooperating with familiar partners or strangers are shown. Paired t test or Mann−Whitney test. E Introducing obstacles did not affect the cooperation index. The CI of mice cooperating without obstacles or with obstacles are shown. Paired t test or Mann−Whitney test. F Cooperative memory last long. The CI of mice on day 21and day 37 are shown. Error bars represent the SEM. Paired t test or Mann−Whitney test. *p < 0.05, ***p < 0.001, n.s. = not significant. **Fig. S2.** Shuttling synchronization was higher in the coop group. A Photo of the cooperation box illustrating video-based analysis. Colorful dots on the mice indicate the neck and body coordinates. Tags on the left indicate area segmentation of the training box. Axis and tags on the right indicate body locations. B Example of synchronous and asynchronous shuttle behaviors. The red and blue lines represent the projection of body centers of the two mice on the axis in A. C Shuttling synchronization of the coop group was higher than that of the non-coop group. One-way ANOVA test. Error bars indicate the SEM. ***p < 0.001. D Shuttling synchronization was positively correlated with the cooperation index. Dots with different shapes indicate the data of six training pairs on various training days. The heat level indicates the number of training days. E Schematic diagram of the definition of social contact. **Fig. S3.** Communications between partners are important. A Cooperation index decreased when mice cooperated with non-trained partners. Paired t test. B Cooperation index decreased during the light-off test. Paired t test or Mann-Whitney test. Error bars represent SEM. *p < 0.05, **p < 0.01. Fig. S4. Cooperation test results of animals to be sacrificed for whole-brain imaging of c-Fos antibody expression and activity trace mapping. Results of both the coop and control groups are shown. Error bars represent SEM. Mann−Whitney test. ***p < 0.001.

**Additional file 2: Video S1.** Example of lever-pressing of mice in the coop group, related to Fig. 1D. Video playback speed is 1 × .

**Additional file 3: Video S2. **Example of lever-pressing of mice in the non-coop group, related to Fig. 1D. Video playback speed is 1 × .

**Additional file 4: Video S3.** Example of social contact behavior, related to Fig. 1E and F. Video playback speed is 0.5 × .

**Additional file 5: Video S4.** Example of waiting behavior, related to Fig. 1H and I. Video playback speed is 0.5 × .

**Additional file 6: Video S5. **Example of cooperative behavior when mice cooperated with trained and non-trained partners, related to Figure S3A. Video playback speed is 1 × .

## Data Availability

The datasets obtained and/or analyzed in the current study are available from the corresponding author on reasonable request.

## Abbreviations

VISoR: High-speed volumetric imaging with synchronized on-the-fly-scan and readout
CI: Cooperation index
FRP: Frontal pole
MO: Somatomotor areas
SS: Somatosensory areas
GU: Gustatory areas
VISC: Visceral area
AUD: Auditory areas
VIS: Visual areas
ACA: Anterior cingulate area
PL: Prelimbic area
ILA: Infralimbic area
ORB: Orbital area
AI: Agranular insular area
RSP: Retrosplenial area
PTLp: Posterior parietal association areas
TEa: Temporal association areas
PERI: Perirhinal area
ECT: Ectorhinal area
OLF: Olfactory areas
HIP: Hippocampal region
RHP: Retrohippocampal region
CLA: Claustrum
EP: Endopiriform nucleus
LA: Lateral amygdalar nucleus
BLA: Basolateral amygdalar nucleus
BMA: Basomedial amygdalar nucleus
PA: Posterior amygdalar nucleus
STRd: Striatum dorsal region
STRv: Striatum ventral region
LSX: Lateral septal complex
sAMY: Striatum-like amygdalar nuclei
PALd: Pallidum dorsal region
PALv: Pallidum ventral region
PALm: Pallidum medial region
PALc: Pallidum caudal region
DORsm: Thalamus sensory-motor cortex related
DORpm: Thalamus polymodal association cortex related
PVZ: Periventricular zone
PVR: Periventricular region
MEZ: Hypothalamic medial zone
LZ: Hypothalamic lateral zone
ME: Median eminence
SCs: Superior colliculus sensory related
IC: Inferior colliculus
SNr: Substantia nigra reticular part
VTA: Ventral tegmental area
RR: Midbrain reticular nucleus retrorubral area
MRN: Midbrain reticular nucleus
SCm: Superior colliculus motor related
PAG: Periaqueductal gray
PRT: Pretectal region
CUN: Cuneiform nucleus
RN: Red nucleus
SNc: Substantia nigra compact part
PPN: Pedunculopontine nucleus
RAmb: Midbrain raphe nuclei
P-sen: Pons sensory related
P-mot: Pons motor related
P-sat: Pons behavioral state related
MY-sen: Medulla sensory related
MY-mot: Medulla motor related
MY-sat: Medulla behavioral state related
