# Supplementary material for: A new paradigm of learned cooperation reveals extensive social coordination and specific cortical activation in mice
Source: Mol Brain. 2023 May 11;16:40. doi: 10.1186/s13041-023-01032-y (PMC10176944; doi:10.1186/s13041-023-01032-y)
Supplement: Supplementary file 1 — Additional file 1: Fig. S1. Mice were able to learn the cooperative lever-pressing task. A Schematic diagram of the cooperative training procedure. B, C Mice were able to learn the individual lever-pressing task. B The total press number during pretraining increased. There was no significant difference between the coop and non-coop groups across the 3 pretraining days. One-way ANOVA test. C The total lick number during pretraining increased. There was no significant difference between the coop and non-coop groups across the 3 pretraining days. One-way ANOVA test. D Swapping partners did not affect the cooperation index. The CI of mice cooperating with familiar partners or strangers are shown. Paired t test or Mann−Whitney test. E Introducing obstacles did not affect the cooperation index. The CI of mice cooperating without obstacles or with obstacles are shown. Paired t test or Mann−Whitney test. F Cooperative memory last long. The CI of mice on day 21and day 37 are shown. Error bars represent the SEM. Paired t test or Mann−Whitney test. *p < 0.05, ***p < 0.001, n.s. = not significant. Fig. S2. Shuttling synchronization was higher in the coop group. A Photo of the cooperation box illustrating video-based analysis. Colorful dots on the mice indicate the neck and body coordinates. Tags on the left indicate area segmentation of the training box. Axis and tags on the right indicate body locations. B Example of synchronous and asynchronous shuttle behaviors. The red and blue lines represent the projection of body centers of the two mice on the axis in A. C Shuttling synchronization of the coop group was higher than that of the non-coop group. One-way ANOVA test. Error bars indicate the SEM. ***p < 0.001. D Shuttling synchronization was positively correlated with the cooperation index. Dots with different shapes indicate the data of six training pairs on various training days. The heat level indicates the number of training days. E Schematic diagram of the definition of s [file 13041_2023_1032_MOESM1_ESM.docx]

Additional file 1

**A new paradigm of learned cooperation reveals extensive social coordination and specific cortical activation in mice**

Ke-Ming Zhang, Yan Shen, Chun-Hui Jia, Hao Wang, Guo-Qiang Bi, Pak-Ming Lau

*Correspondence: [plau@ustc.edu.cn](mailto:plau@ustc.edu.cn) or gqbi@ustc.edu.cn

**This file includes:**

Materials and Methods

Figure S1

Figure S2

Figure S3

Figure S4

**Materials and Methods**

**Animal**

The animals used in the study were adult male C57BL/6J mice (SiPeiFu, China). The mice were group-housed under a 12-hour light/dark cycle (8:00-20:00). Before the training procedure, all mice had free access to food and water. Mice were deprived of water 16 hours before training, and their body weight was checked daily. If their body weight was less than 85% of their initial body weight, they were free to drink water for 3 minutes after each day of training. All animal procedures were approved by the Animal Care and Use Committees of the University of Science and Technology of China.

**Behavioral apparatus**

An acrylic box, 30 × 20 × 40 cm in length, width and height, was separated by a partition wall into two small chambers, each with a lever at one end and a lickometer at the other end. Infrared sensors were used to detect presses and licks from the lever and the lickometer, respectively. The lickometer was connected to a peristaltic pump. An Arduino board received data from the infrared sensors in real time and determined whether to control the peristaltic pump to supply water to the lickometer [1]. A script is written on the computer side using Bonsai for communicating with the Arduino and storing the behavior videos [2].

**Training of cooperative behavior**

For cooperative behavior, the training process was divided into two phases. The first phase was pretraining, which was conducted in the same behavior box for three days, and this phase was used to train mice to learn the individual lever-pressing task with a white unpunched partition wall. The mice were trained twice a day. One was from 9:00 a.m. to 11:00 a.m. and the other was 9:00 p.m. to 11:00 p.m. On the first, second and third days of pretraining, the mice received 50 μL, 30 μL and 20 μL of water per press, respectively; the training was terminated when the mice obtained more than 500 μL of water in one session or the session lasted for 1 hour.

On the first day of cooperative training, the mice were divided into cooperative and non-cooperative groups, and the partition wall was replaced with transparent windowed one; the cooperative group was trained to press cooperatively, while the non-cooperative group continued the pretraining rules, and both groups were still trained twice a day for 10-21 days; for the cooperative group, if both levers were pressed and the time difference was within the time window, the mice were allowed to receive rewards. For the non-cooperative group, the mice could receive a 10 μL water reward for lever pressing alone. The difficulty of cooperative training was gradually increased: on the 1st day, the time window was 1 s. When the mice cooperated within 1 s, they received 50 μL of water; if the mice received 500 μL of water within 30 min in a session, the reward for the next session was changed to 30 μL and 20 μL in turn; if not, the reward value was maintained; after changing to 20 μL, if the mice received 500 μL of water within 30 min, the cooperative time window for the next session was shortened to 0.5 s; if mice could obtain 500 μL reward within 30 min under the time window of 0.5 s, the amount of each reward for the next session was changed to 10 μL (**Fig. S1A**). The paired mice were always put back to the same homecage after training.

The success rate of cooperation training as assessed by **the cooperation index** (CI, see below for definition) of the trained animal in the last training day and during positive control tests after training was greater than zero. Among all 40 mice in the coop group, 38 mice achieved this criterion (2 mice in Fig. S1D did not achieve this criterion). The model success rate is 95%.

**Blocking social contact test**

After the mice learned to cooperate, this test was performed by replacing the windowed partition with an unpunched partition and turning off the light (**Fig. 1M**). The time window for reward was 0.5 s. The reward value was 10 μL. The test lasted for 20 min, and the cooperation index was calculated.

**Light-off test**

After the mice learned to cooperate, this test was performed with light turned off (**Fig. S3B**). The time window for reward was 0.5 s. The reward value was 10 μL. The test lasted for 20 min, and the cooperation index was calculated.

**Partner swapping test**

After the mice learned to cooperate, two mice from different cooperative training pairs (**Fig.S1D**) or from cooperatively trained and non-cooperatively trained groups respectively (**Fig. S3A**) were placed into the same box for the cooperation test. The time window for reward was 0.5 s. The reward value was 10 μL. The test lasted for 20 min, and the cooperation index was calculated.

**Obstacle test**

Mice were tested after they learned to cooperate by placing an obstacle into the training box, obstructing the mice from shuttling freely between the lever and the lickometer. The time window for reward was 0.5 s. The reward value was 10 μL. The test lasted for 20 min, and the cooperation index was calculated.

**Long-term memory test**

The cooperation test was stopped after the mice learned to cooperate. The mice were placed into the homecage with free access to food and water. After 15 days, water was again deprived for 16 hours, after which the cooperative press was tested. The time window for reward was 0.5 s. The reward value was 10 μL. The test lasted for 20 min, and the cooperation index was calculated.

**Cooperation index**

Based on the behavioral data sent by Arduino, a customized MATLAB script was used to extract each press and lick of each mouse, and to calculate the number of cooperative presses, total presses and the ratio of cooperative presses. For the cooperation index (CI), the ratio of shuffled cooperative presses and total presses was calculated, and the difference between the real ratio and the 95% upper limit of the shuffled ratio was defined as the CI (**Fig. 1D**). For shuffling the data, based on the time sequence and duration of the real presses and licks of each mouse in each session, only the time sequence of all presses and licks was shuffled without changing the duration distribution; each session was shuffled 1000 times.

**Social contact and wait behavior classification**

The analysis was based on behavioral videos using a deep learning method. First, we selected a total of 200 frames from different behavioral videos using DeepLabCut to form a training set, labeled the neck and body center of each mouse in each image, and trained the neural network to recognize the neck and body of mice [3]; after that, we segmented the behavior box into 3 areas: press area, middle area and lick area. The press area and lick area were defined as the area 10 cm in front of the wall where the lever was located and the area 10 cm in front of the wall where the water pipe was located; the middle area is the area 10 cm in the middle (**Fig. S2A**).

The body coordinates of the mice were analyzed using a customized MATLAB script. For the body angle, the center of the body was taken as the origin, and a horizontal line was made in the direction of the lever; for the mice on the lower side of the video, a horizontal line was rotated counterclockwise by one turn for 0-360°, and for the mice on the upper side, a horizontal line was rotated clockwise by one turn for 0-360°, and the angle between the body and neck line of the mice and the horizontal line was defined as the body angle (**Fig. S2E**); for the neck spacing, the straight-line distance between the neck centers of two mice was calculated.

Based on the neck and body positions of the mice, social contact and waiting behaviors were classified as follows: social contact (the neck distance between the two mice was no more than 80 mm, the body angles were in the range of 5-175°, and the two mice were not licking or pressing, **Fig. 1E, Fig. S2E, video S3**) and wait (the mouse was out of the lick area, closer to lever, not social contacting and not pressing; once its partner entered the press area, the mouse pressed the lever within 2 s and left the press area within 2 s after pressing; before press, the duration of the mouse body facing to the opposite side, body angle between 5° and 175°, exceeded 0.5 s; the waiting duration was not less than 1 s. **Fig. 1H, video S4**).

**Shuttling synchronization**

The shuttling synchronization is defined as the Pearson’s r value of the projection of body centers on the long axis of the training box (**Fig. S2A**). For comparing of shuttling synchronization between the coop and non-coop groups, the mean Pearson’s r of each group during training days 17, 19 and 21 was calculated (**Fig. S2C**).

**Correlation analysis**

For the correlation analysis of social contact, wait, shuttling synchronization and CI, the Pearson r was calculated (**Fig. 1G, J, Fig. S2D**).

**Immunohistochemistry**

Mice were divided into coop (n = 12) and control (n = 6) groups, undergoing cooperative training and individual training, respectively (**Fig. S4**). On the day of sampling, mice were anesthetized 1.5 hours after cooperation testing for perfusion.

The mice were anesthetized with 1% sodium pentobarbital solution (80 mg/kg) and started to perfuse 20 mL of 37 ℃ and 4 ℃ PBS solution at a rate of 20 mL/min, respectively, and 20 mL of 4% hydrogel monomer solution (HMS) at a rate of 20 mL/min; after the perfusion was completed, brain tissue was removed and placed in 20 mL of 4% HMS and post-fixed at 4 ℃ for 3 days; brain tissue was then placed in a 1:1 mixture of 20% BSA and 4% HMS solution and polymerized at 37 ℃ for 4 hours; after that, brain tissue was sliced into 300 μm-thick coronal sections using a vibrating microtome (WPI) and permeabilized with 0.5% PBST at 37 ℃ for 22 hours, followed by three washes with PBS; c-Fos primary antibody solution (c-Fos (9F6) Rabbit mAb, 1:2000) was incubated for 38 h at 4 °C, followed by 3 washes with PBS, then secondary antibody solution (Cy^TM^3, 1:250) was incubated for 18 h at 4 °C, followed by 3 washes with PBS; the sections were imbibed with refractive index-matched solution (RI = 1.52, containing 50% iodophor, 23% urea, 11% triethanolamine, 16% ultrapure water) until completely transparent; the sections were finally imaged at 1 × 1 × 5 μm^3^ voxel resolution using the VISoR system, followed by whole-brain 3D reconstruction at 4 × 4 × 4 μm^3^ using custom software [4].

**c-Fos counting**

For the identification and statistics of c-Fos signals, the main steps included the identification of cells expressing c-Fos using Ilastik [5], counting the number of c-Fos positive cells in each brain region using Freesia [4], and constructing a linear model to test the significance between groups [6].

For the identification of c-Fos positive cells using Ilastik, the reconstructed 4 μm-thick projection images were first merged into 25 μm-thick projection images using ImageJ, and then the two 25 μm images of the c-Fos signal channel and the reference channel were merged into one bicolor image. Four bicolor images from each mouse brain were uniformly selected as the training set, and the network was trained to recognize the c-Fos signal using Ilastik's two-step method: the first step was pixel recognition, and the network was trained to recognize the pixels by manually labeling the c-Fos signal and background noise in each training image separately; after training, the network was checked to recognize the c-Fos signal and noise in the unlabeled area, and if it could not distinguish the c-Fos signal well, more c-Fos positive pixels of the training images were labeled, and these steps were repeated until the network was able to recognize the c-Fos signal in all training images. The second step was cell recognition. Importing the neural network trained in the first step and several images into Ilastik, using the neural network to recognize the c-Fos signal in them, and adjusting the threshold of cell recognition until the software was able to recognize individual cells. After these two steps were completed, all the images were imported into the first step of the workflow, and the trained neural network was used to identify the c-Fos pixels. Then the results of the first step were imported into the second step to identify all cells expressing c-Fos. Finally, Ilastik outputs the spatial coordinates, grayscale, and size of all identified cells [5]. This information was imported into Freesia. The locations of the brain regions automatically delineated by Freesia were manually checked and adjusted. The counting function of Freesia was used to output the c-Fos expression counts and density for all brain regions of each mouse [4].

**Data statistics**

The statistical analysis of behavioral data was performed using one-way ANOVA, paired t or Mann−Whitney test. Behavioral data are expressed as the means ± SEM. These statistical analyses were conducted in Origin (OriginLab). *p* < 0.05 was defined as statistically significant.

For the statistics of c-Fos data, a linear regression model Y = βX + α was established using a custom MATLAB script, assuming that the c-Fos counts conform to a negative binomial distribution [6, 7], where Y is a matrix composed of the c-Fos expression density of all brain regions in all mice, the total number of rows of the matrix is equal to the total number of mice, and the total number of columns of the matrix is equal to the number of brain regions. X is a binary vector consisting of grouping tags of all mice. The mice in the coop group were labeled 1. The mice in the control group were labeled as 0. The total number of elements in X was the same as the total number of mice, and the order of the elements corresponds to Y. β is the model coefficient, representing the direction of change of Y with the change of X, and α is a constant. A linear model was constructed for each column in Y with respect to X. For each linear model, a hypothesis test was performed to determine whether the coefficient β was 0. The p value was corrected for false discovery rate (FDR) using the Benjamini−Hochberg method [8].

**References**

1. Isett BR, Feasel SH, Lane MA, Feldman DE. Slip-Based Coding of Local Shape and Texture in Mouse S1. Neuron. 2018;97(2):418-33 e5.

2. Lopes G, Bonacchi N, Frazao J, Neto JP, Atallah BV, Soares S, et al. Bonsai: an event-based framework for processing and controlling data streams. Front Neuroinform. 2015;9:7.

3. Mathis A, Mamidanna P, Cury KM, Abe T, Murthy VN, Mathis MW, et al. DeepLabCut: markerless pose estimation of user-defined body parts with deep learning. Nat Neurosci. 2018;21(9):1281-9.

4. Wang H, Zhu Q, Ding L, Shen Y, Yang C-Y, Xu F, et al. Scalable volumetric imaging for ultrahigh-speed brain mapping at synaptic resolution. Natl Sci Rev. 2019;6(5):982-92.

5. Berg S, Kutra D, Kroeger T, Straehle CN, Kausler BX, Haubold C, et al. Ilastik: interactive machine learning for (bio) image analysis. Nat Methods. 2019;16(12):1226-32.

6. Kim Y, Venkataraju KU, Pradhan K, Mende C, Taranda J, Turaga SC, et al. Mapping social behavior-induced brain activation at cellular resolution in the mouse. Cell Rep. 2015;10(2):292-305.

7. O'Hara R, Kotze J. Do not log-transform count data. Methods Ecol Evol. 2010:118-122.

8. Benjamini Y, Hochberg Y. Controlling the false discovery rate: a practical and powerful approach to multiple testing. J R Stat Soc Ser B Methodol. 1995;57(1):289-300.


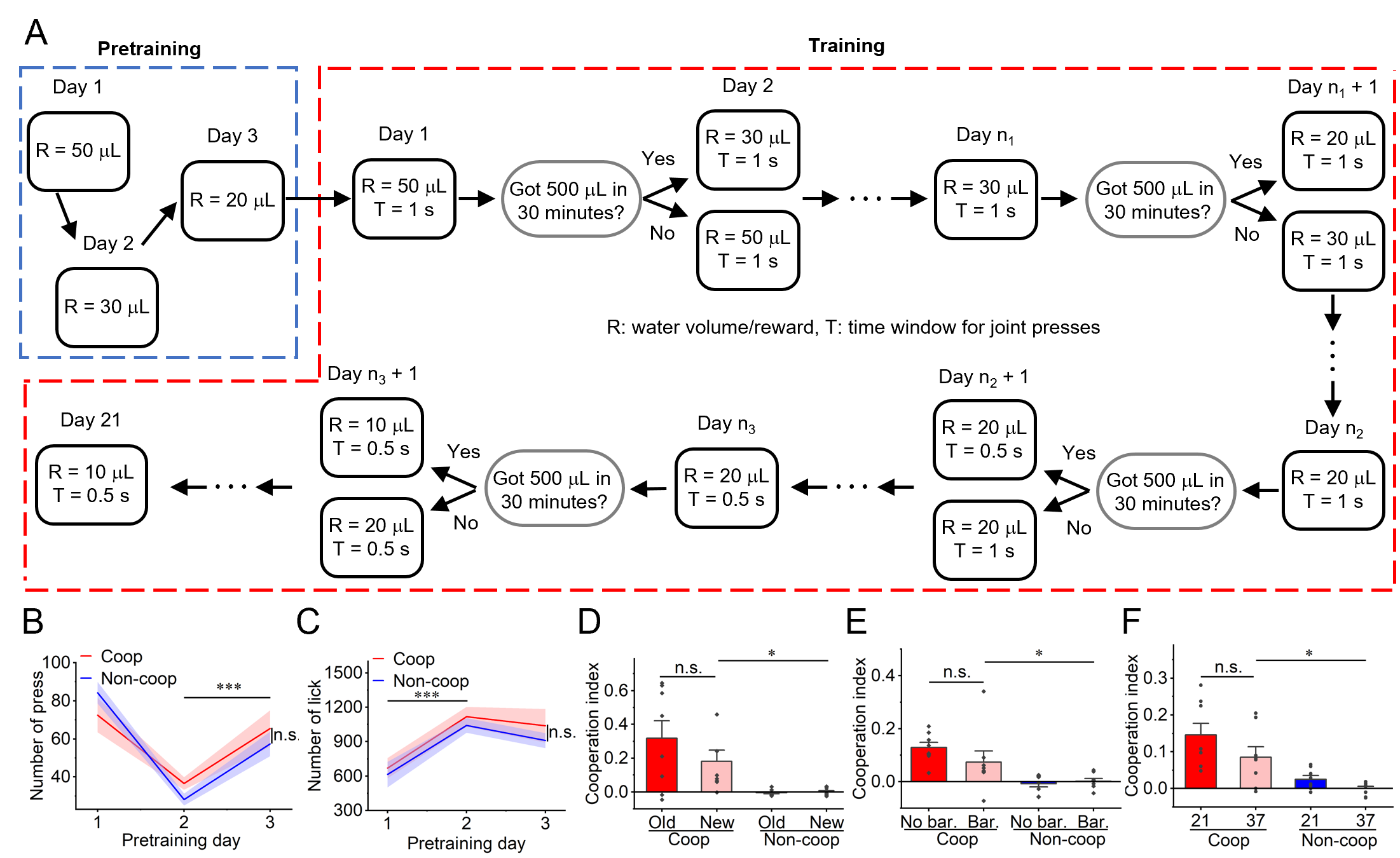


**Fig. S1** **Mice were able to learn the cooperative lever-pressing task.**

**A** Schematic diagram of the cooperative training procedure. **B, C** Mice were able to learn the individual lever-pressing task. **B** The total press number during pretraining increased (day 3 vs day 2, n = 36, paired t test). There was no significant difference between the coop (n = 20) and non-coop (n = 16) groups across the 3 pretraining days. One-way ANOVA test. **C** The total lick number during pretraining increased (day 2 vs day 1, n = 36, paired t test). There was no significant difference between the coop (n = 20) and non-coop (n = 16) groups across the 3 pretraining days. One-way ANOVA test. **D** Swapping partners did not affect the cooperation index (CI). The CI of mice cooperating with familiar partners (Old, n = 8) or strangers (New, n = 8) are shown. Paired t test (old_coop_ vs new_coop_) or Mann−Whitney test (new_coop_ vs new_non-coop_). **E** Introducing obstacles did not affect the cooperation index. The CI of mice cooperating without obstacles (No bar., n = 8) or with obstacles (Bar., n = 8) are shown. Paired t test (no bar._coop_ vs bar._coop_) or Mann−Whitney test (bar._coop_ vs bar_non-coop_). **F** Cooperative memory last long. The CI of mice on day 21 (the last training day, n = 8) and day 37 (after 15 days of training interruption and free access to water, n = 8) are shown. Error bars represent the SEM. Paired t test (21_coop_ vs 37_coop_) or Mann−Whitney test (37_coop_ vs 37_non-coop_). *p < 0.05, ***p < 0.001, n.s. = not significant.


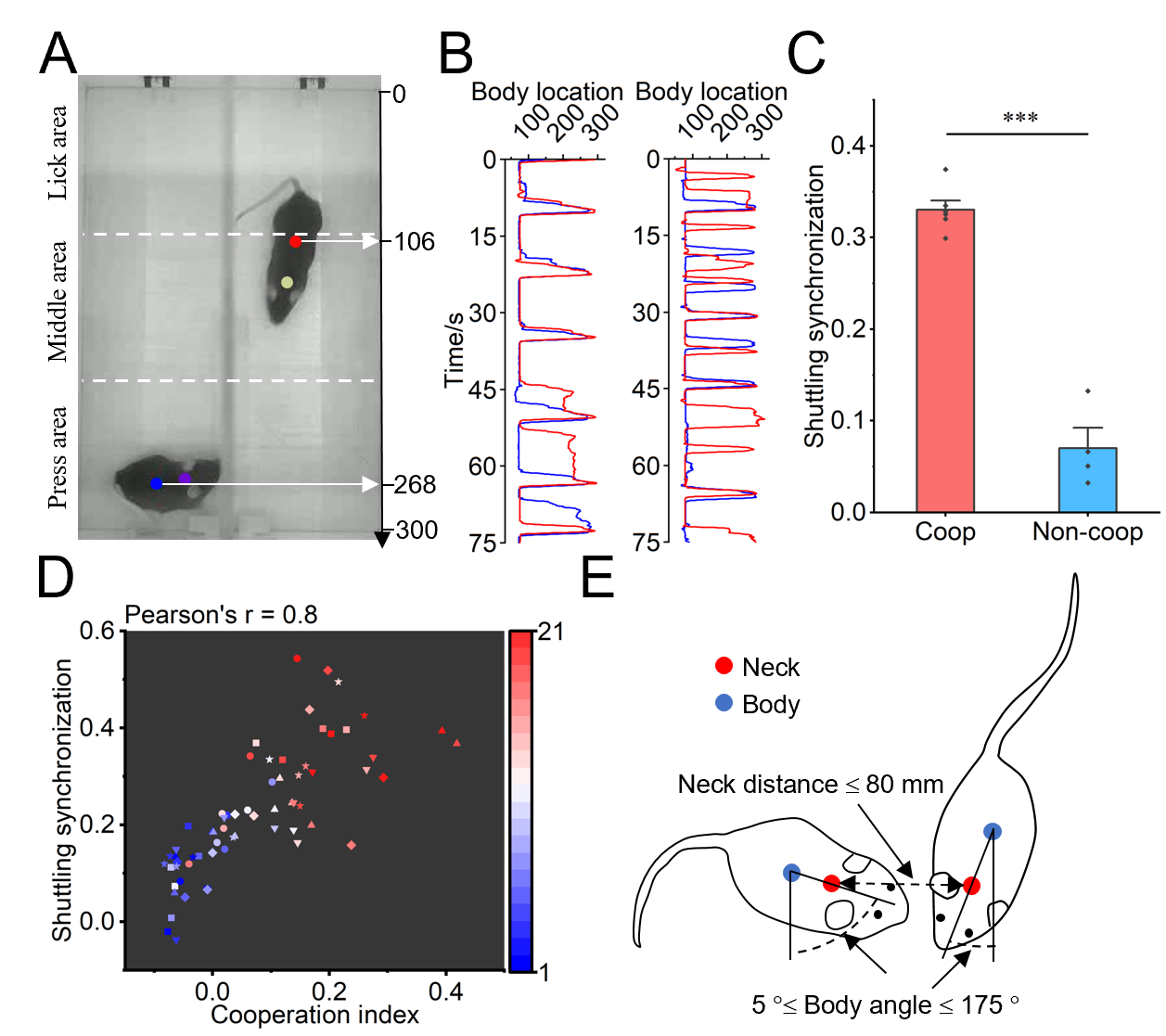


**Fig. S2 Shuttling synchronization was higher in the coop group.**

**A** Photo of the cooperation box illustrating video-based analysis. Colorful dots on the mice indicate the neck and body coordinates. Tags on the left indicate area segmentation of the training box. Axis and tags on the right indicate body locations. **B** Example of synchronous (left, coop group) and asynchronous (right, non-coop group) shuttle behaviors. The red and blue lines represent the projection of body centers of the two mice on the axis in **A**. **C** Shuttling synchronization of the coop group (mean of Pearson’s r of body locations of each pair during training days 17, 19 and 21, n = 6 pairs) was higher than that of the non-coop group (mean of Pearson’s r of body locations of each pair during training days 17, 19 and 21, n = 4 pairs). One-way ANOVA test. Error bars indicate the SEM. ***p < 0.001. **D** Shuttling synchronization was positively correlated with the cooperation index. Dots with different shapes indicate the data of six training pairs on various training days. The heat level indicates the number of training days. **E** Schematic diagram of the definition of social contact.


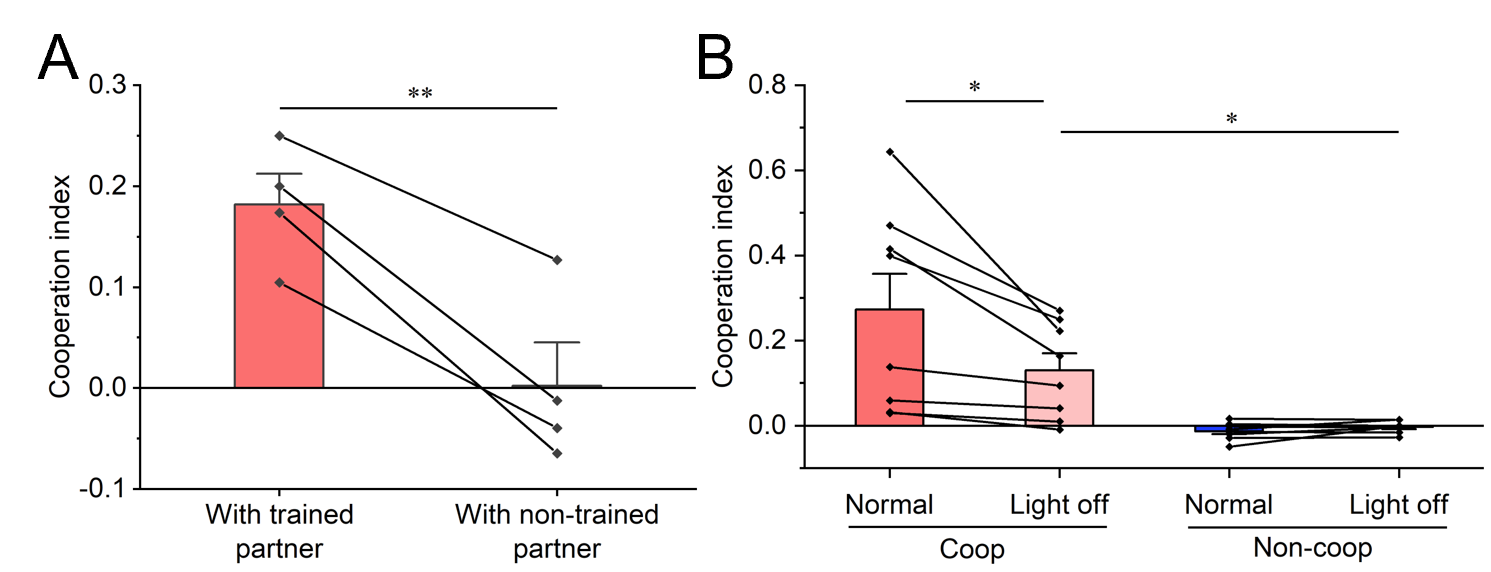


**Fig. S3 Communications between partners are important**

**A** Cooperation index decreased when mice cooperated with non-trained partners. Paired t test (n = 4). **B** Cooperation index decreased during the light-off test. Paired t test (normal condition of coop group vs light-off condition of coop group, n = 8) or Mann-Whitney test (light-off condition of coop group vs light-off condition of non-coop group, n = 8). Error bars represent SEM. *p < 0.05, **p < 0.01.


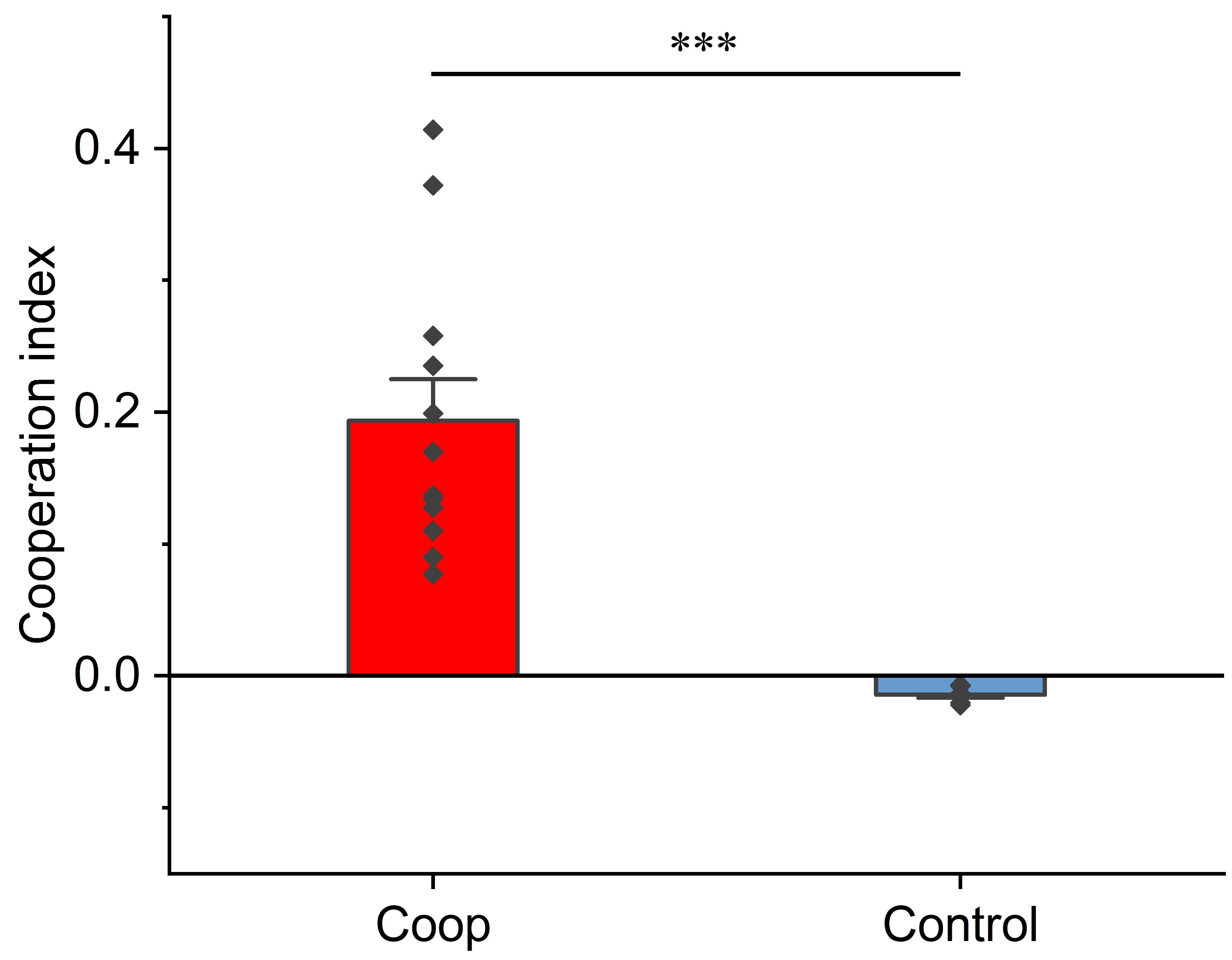


**Fig. S4 Cooperation test results of animals to be sacrificed for whole-brain imaging of c-Fos antibody expression and activity trace mapping.**

Results of both the coop (n = 12) and control (n = 6) groups are shown. Error bars represent SEM. Mann−Whitney test. ***p < 0.001.
